# Anomalous Systemic Arterial Supply to the Basal Segments with an Aneurysmal Aberrant Artery Showing Advanced Wall Fragility: A Case Report and Literature Review

**DOI:** 10.70352/scrj.cr.25-0823

**Published:** 2026-03-20

**Authors:** Shun Yorimori, Shigeki Suzuki, Kenta Shida, Kosuke Sugino, Takahiro Suzuki, Yu Okubo, Hirofumi Haida, Kyohei Masai, Kaoru Kaseda, Yutaka Kurebayashi, Kenichi Hashizume, Hideyuki Shimizu, Keisuke Asakura

**Affiliations:** 1 Division of Thoracic Surgery, Department of Surgery, Keio University School of Medicine, Tokyo, Japan; 2 Division of Cardiovascular Surgery, Department of Surgery, Keio University School of Medicine, Tokyo, Japan; 3 Department of Pathology, Keio University School of Medicine, Tokyo, Japan

**Keywords:** anomalous systemic arterial supply to the basal segments, thoracic endovascular aortic repair, aneurysmal aberrant artery, lobectomy

## Abstract

**INTRODUCTION:**

Anomalous systemic arterial supply to the basal segments (ASABS) is a rare congenital pulmonary anomaly. Considering the associated complications of pulmonary hypertension or hemoptysis, surgical lung resection is recommended. However, there is a lack of standardized surgical management guidelines. We report a case of ASABS with an aneurysmal aberrant artery, in which imaging findings suggested an increased risk of rupture. Thoracic endovascular aortic repair (TEVAR) was first performed to achieve inflow control; this strategy provided a secure setting for subsequent pulmonary resections.

**CASE PRESENTATION:**

A 56-year-old woman presented with an abnormal chest shadow. Contrast-enhanced chest CT revealed an aberrant artery arising from the descending aorta supplying the left basal segment with an irregular aneurysm measuring 33 mm. Non-contrast chest CT demonstrated intraluminal hyperattenuation consistent with acute thrombosis, raising concerns regarding an increased risk of rupture. The absence of a normal pulmonary arterial supply to the basal segment and normal bronchial and pulmonary venous anatomy confirmed the diagnosis of ASABS. Given the radiologic findings suggestive of an increased risk of rupture of an aberrant artery, preoperative TEVAR was performed to control the inflow to the aberrant artery. After 18 days, video-assisted left lower lobectomy with division of the aberrant artery was performed safely. The postoperative course was uneventful, and the patient was discharged 10 days after surgery. Pathology revealed disruption of the elastic fibers and thinning of the media, supporting aneurysmal degeneration at the risk of rupture. Contrast-enhanced CT performed 6 months after surgery confirmed the absence of an endoleak or stump aneurysm.

**CONCLUSIONS:**

The TEVAR-first approach for inflow control enabled safe pulmonary resection in ASABS with an aneurysmal aberrant artery. To the best of our knowledge, this is the first reported case for this condition where histopathological findings suggested an increased risk of rupture. Further investigation is needed to determine which patients benefit the most from this strategy and to understand the long-term outcomes.

## Abbreviations

ASABS
: anomalous systemic arterial supply to the basal segments
EVG
: Elastica van Gieson
TEVAR
: thoracic endovascular aortic repair

## INTRODUCTION

ASABS is an uncommon congenital pulmonary anomaly in which the basal lung lacks a pulmonary arterial branch, yet retains normal bronchial anatomy and pulmonary venous drainage. This segment receives systemic arterial perfusion from the descending aorta.^1,2)^ A left-to-left shunt under systemic pressure can cause pulmonary hypertension and hemoptysis.^3,4)^ Therefore, surgery is generally recommended. However, standardized guidelines for surgical treatment have not yet been established. Some of the key unresolved issues are establishing the extent of pulmonary resection based on the perfused territory of the aberrant artery and methods for managing the aberrant artery.

This report describes a case of ASABS showing an aneurysmal aberrant artery with imaging features compatible with an increased risk of rupture, including an irregular saccular aneurysm and intraluminal hyperattenuation on non-contrast CT. We performed lobectomy safely because TEVAR was first performed to achieve inflow control. Notably, the resected specimen showed histopathological findings suggestive of advanced wall fragility and an increased risk of rupture, which, to the best of our knowledge, has not been explicitly described in previous ASABS reports. Therefore, we report this rare case and discuss its clinical implications by reviewing the relevant literature.

## CASE PRESENTATION

A 56-year-old woman was referred to our hospital with an abnormal shadow in the left lower lung field detected on routine chest radiography. The patient was asymptomatic and in good general health. She had hypertension and hyperlipidemia. The patient had a 10-pack-year smoking history. Physical examination revealed no remarkable findings, and all laboratory parameters were within normal limits. The patient exhibited normal pulmonary function. Cardiac function was normal and transthoracic echocardiography revealed no evidence of right heart overload.

**Fig. 1:**
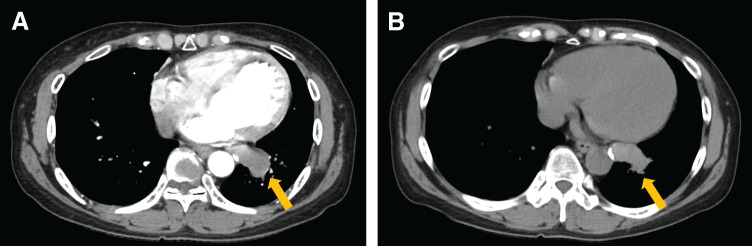
Preoperative chest CT. (**A**) Contrast-enhanced chest CT showing a 33 mm, irregularly shaped saccular aneurysm arising from an aberrant artery supplying the left basal segment (arrow). (**B**) Non-contrast chest CT showing a hyperdense lumen within the aneurysm (arrow), suggestive of acute thrombus formation.

**Fig. 2:**
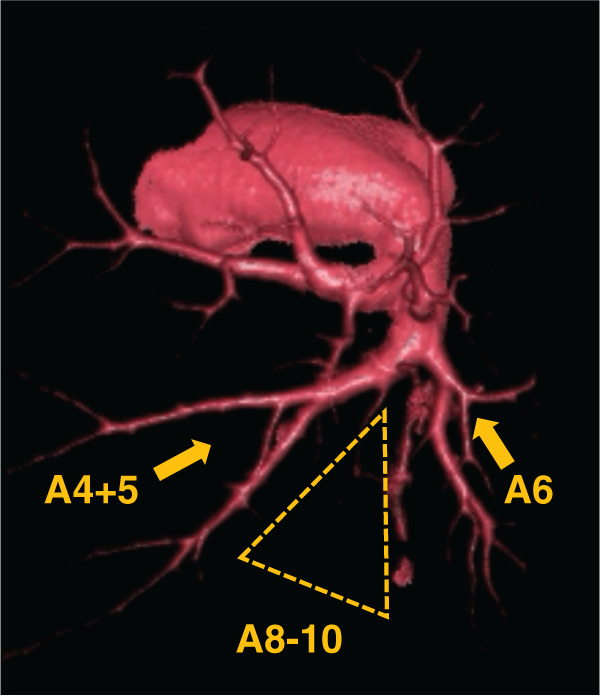
Preoperative 3D CT angiography of the left pulmonary artery. This figure demonstrates normal branches to A4+5 and A6 from the left pulmonary artery (arrows) without basal segmental branches (area enclosed by the dotted line).

**Fig. 3:**
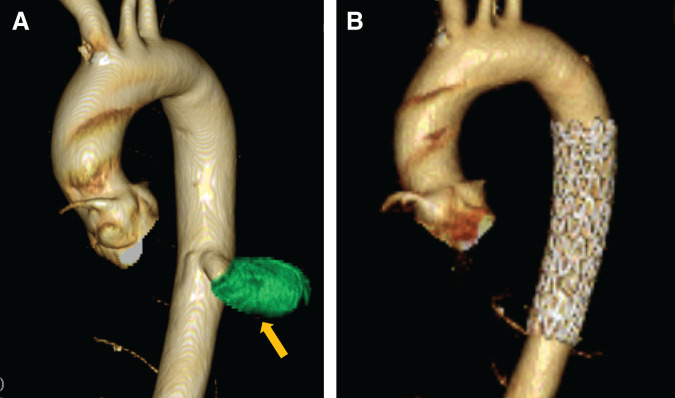
3D CT angiography performed before and after TEVAR. (**A**) This figure shows an irregular aneurysm arising from the descending aorta (arrow). (**B**) This figure demonstrates successful flow exclusion within the aberrant artery and its aneurysm. TEVAR, thoracic endovascular aortic repair

**Fig. 4:**
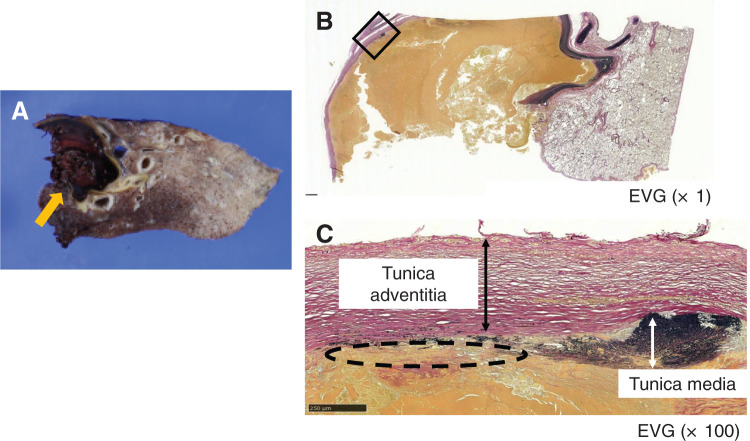
Macroscopic and histologic findings. (**A**) The resected lung specimen showing a markedly dilated aberrant artery filled with organizing thrombus (arrow). (**B**, **C**) EVG at low magnification (×1) and high magnification (×100) revealed focal loss of elastic fibers and marked fibrous thickening of the tunica adventitia, leaving only a fibrotic outer wall. EVG, Elastica van Gieson

Chest radiography revealed an enlarged shadow in the left lower lung field (**Supplementary Fig. S1**). Contrast-enhanced chest CT revealed an aberrant artery flowing from the descending aorta into the left basal segment, and an irregular saccular aneurysm measuring 33 mm in diameter (**Fig. 1A**). Anatomy of the bronchi and pulmonary veins was normal. Non-contrast chest CT of the aneurysm lumen showed partial calcification and high attenuation suggesting acute thrombosis (**Fig. 1B**). Thus, the formation of an irregular saccular aneurysm and acute thrombosis raised concern for an increased risk of aneurysmal rupture. 3D reconstruction CT (3D CT) demonstrated that A4+5 and A6 branched off from the pulmonary artery trunk. However, branches to the basal segments were absent (**Fig. 2**). We made a preoperative diagnosis of ASABS with an aneurysmal aberrant artery at high risk of rupture. Therefore, division of the aberrant artery and pulmonary resection were deemed appropriate. Although the patient was asymptomatic, these findings indicated a substantial risk of aneurysmal rupture during the waiting period and at the time of surgery. Given the large caliber and aneurysmal degeneration of the aberrant artery, we concluded that surgical division using a linear stapler alone would not provide sufficient hemodynamic control, and might be associated with uncontrollable intraoperative bleeding. Thus, we selected a TEVAR-first strategy to achieve secure inflow exclusion before pulmonary resection on a semi-urgent basis, and a two-stage strategy with subsequent pulmonary resection. The use of TEVAR for this disease is beyond the standard indications in Japan. However, we performed TEVAR as an off-label intervention and obtained written informed consent from the patient because a high risk of rupture was strongly suspected based on imaging findings. After identifying the artery of Adamkiewicz and its relationship to the aberrant arterial origin on preoperative CT, TEVAR using a thoracic aortic stent graft was performed through the right femoral artery under general anesthesia. We deployed a Conformable GORE TAG Thoracic Endoprosthesis (cTAG; W.L. Gore & Associates, Flagstaff, AZ, USA), tapered 31–26 × 100 mm. To minimize the risk of spinal cord ischemia, a short stent graft (100 mm) was selected to limit the coverage of the descending aorta, spanning from Th7 to Th12. Particular attention was paid to the relationship between the landing zones and intercostal arteries, including the artery of Adamkiewicz, on preoperative imaging. Intraoperative motor evoked potential monitoring was performed, and the mean arterial pressure was maintained above 80 mmHg throughout the procedure. Intraoperative angiography confirmed complete exclusion of the aberrant artery without evidence of endoleak. After TEVAR, no blood flow was observed in the aberrant artery (**Fig. 3A**, **3B**). The interval of 18 days before pulmonary resection was chosen to confirm stable inflow exclusion and the absence of endoleak after TEVAR, while avoiding excessive delay given the concern for a high risk of rupture. Follow-up contrast-enhanced CT was obtained to assess for reperfusion and endoleak. Left lower lobectomy with division of the aberrant artery was performed 18 days after the TEVAR. Surgery was performed under general anesthesia with thoracoscopic assistance by placing an 8 cm posterolateral incision in the fifth intercostal space and inserting a scope into the eighth intercostal space. An aberrant artery caudal to the inferior pulmonary vein was confirmed (**Supplementary Fig. S2A**). As the aberrant artery was firm on palpation, thrombotic occlusion of the lumen was suspected. Division of the bronchus and vascular structures other than the aberrant artery was performed first, thereby securing adequate working space for subsequent management of the aberrant artery. Firm adhesion was observed between the aberrant artery and inferior pulmonary vein. The adhesion was carefully released and the aberrant artery was exposed (**Supplementary Fig. S2B**). The aberrant artery was substantially hardened due to thrombosis, and transection with a surgical stapler was initially deemed difficult. However, because the thrombosis on the proximal side was less extensive, we considered stapled transection to be feasible and divided the artery at its origin using a linear stapler (DST Series TA) (**Supplementary Fig. S2C**). To prevent postoperative aneurysmal dilatation of the vascular stump, polymer ligating clips (Click’a V, size L, Grena, UK) were applied on the proximal side of the stapled transection line (**Supplementary Fig. S2D**). The operative time was 164 min. The intraoperative bleeding was minimal. The drain was removed on POD 2, and the patient was discharged without complications on POD 10. Contrast-enhanced CT performed 6 months after surgery confirmed the absence of endoleak or stump aneurysm. On postoperative histopathological examination, a dilated aberrant artery was identified within the lung and the lumen was filled with thrombosis (**Fig. 4A**). Furthermore, EVG staining of the aberrant artery demonstrated a loss of elastic fibers associated with marked dilatation, leaving only fibrotically thickened adventitia (**Figs. 4B**, **4C**). These findings are characteristic of a markedly dilated aneurysm and are compatible with advanced wall fragility and an increased risk of rupture of the aberrant artery.

## DISCUSSION

ASABS has historically been classified as Pryce type I within intralobar pulmonary sequestration; however, because there is an absence of sequestered lung, Painter described it as “anomalous systemic arterialization of the lung without sequestration”.^5)^ This disease is characterized by systemic arterial perfusion from the aorta to the basal lung lacking a normal pulmonary arterial branch while exhibiting normal bronchial anatomy and pulmonary venous drainage.^1,2)^ The reported frequency of congenital pulmonary malformations ranges from 0.15% to 6.4%.^6)^ A combination of systemic-pressure perfusion of the pulmonary circulation and a left-to-left shunt may lead to pulmonary hypertension, hemoptysis, and heart failure.^3,4)^ Accordingly, surgical treatment is generally recommended when operative risk is acceptable, even in asymptomatic patients.

Because this condition is rare, definitive guidance on surgical strategies remains elusive. We searched PubMed from 1996 to 2025 using keywords “anomalous systemic arterial supply to the basal segments” and “pulmonary sequestration of Pryce type I,” limiting the results to cases managed surgically. We identified 46 published cases (37 case reports) and analyzed the clinical features along with the present case (**Table 1**).^4,7–42)^ Hemoptysis was the most frequent symptom (51.1%), whereas asymptomatic presentation occurred in 34.0% of patients, which is consistent with the incidental finding in our patient. In most reports, the aberrant artery arose from the descending aorta (89.4%) and the left basal segment constituted the predominant perfusion territory (76.6%).

**Table 1:** Summarized clinical features in previous cases, including our case (38 reports, 47 patients)

Characteristics	Value
Age, median (range)	38 (15–78)
Sex	
Male	26 (55.3)
Female	21 (44.7)
Symptom	
Hemoptysis	24 (51.1)
Chest discomfort	2 (4.3)
Cough	2 (4.3)
Sputum	1 (2.1)
Shortness of breath	1 (2.1)
Pneumonia	1 (2.1)
None	16 (34.0)
Decisive diagnostic test	
3D CT	35 (74.5)
Angiogram	10 (21.3)
Unspecified	2 (4.3)
Aberrant artery origin (overlapping categories)	
Descending thoracic aorta	42 (89.4)
Celiac artery	2 (4.3)
Abdominal artery	2 (4.3)
Unknown	1 (2.1)
Number of aberrant artery, median (range)	1 (1–3)
Diameter of aberrant artery, median (range), mm	16.5 (3–33)
Pulmonary territory supplied by the aberrant artery (overlapping categories)	
Basal segment of left lung	36 (76.6)
Lingular segment of left lung	3 (6.4)
Segment 10 of left lung	2 (4.3)
Basal segment of right lung	2 (4.3)
Segment 10 of right lung	4 (8.5)
Calcification of aberrant artery	3 (6.4)
Aneurysm of aberrant artery	9 (19.1)
Suspected a high risk of rupture	1 (2.1)

Values are presented as n (%) unless otherwise indicated.

Our case shares several features with previously reported ASABS cases, including a left-sided basal perfusion territory and an aberrant artery arising from the descending aorta. However, the differences observed in our patient were that the aberrant artery showed marked aneurysmal dilatation (with a diameter which was at the upper end of the reported range) and the radiological features were suggestive of an increased risk of rupture. Another noteworthy aspect of our case was the histopathological appearance of the aneurysmal aberrant artery. EVG-stained sections demonstrated a focal loss of elastic fibers in the tunica media and marked fibrous thickening of the adventitia leaving only a fibrotic adventitia. Although several reports have described aneurysmal dilatation or wall thinning in aberrant systemic arteries, detailed histopathological findings have rarely been documented. We did not find any previous report explicitly describing such changes suggestive of advanced wall fragility and an increased risk of rupture in ASABS. We believe that these structural alterations reflect advanced wall fragility.

**Fig. 5:**
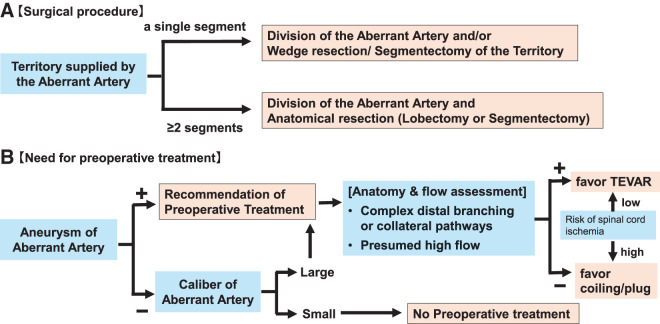
Algorithm of surgical strategy for ASABS. (**A**) Algorithm for determining the extent of lung resection according to the pulmonary territory supplied by the aberrant artery. (**B**) Algorithm for the management of the aberrant artery by the presence of an aneurysm and the caliber of the artery. ASABS, anomalous systemic arterial supply to the basal segment

Based on the operative findings of previous case reports (**Table 2**), we propose two key principles for the surgical strategy in ASABS (**Fig. 5A**, **5B**). **Figure 5A** demonstrated that the extent of pulmonary resection should be determined based on the perfusion territory of the aberrant artery. As shown in **Table 1**, most cases involved perfusion of the entire basal segment; however, segment 10 alone accounted for 12.8% of the perfusion. Several reports have described favorable long-term outcomes with wedge resection or anatomic segmentectomy of the supplied territory when the perfused territory is confined to a segment or smaller area.^33,38)^ Moreover, division of the aberrant artery without lung resection has been reported and could be one of the treatment options in selected patients on the basis of their age and pulmonary function.^40)^ In contrast, retaining a large volume of perfused pulmonary parenchyma may lead to pulmonary congestion or infection.^41)^ Accordingly, when the perfused territory involves two or more segments, anatomical resection aimed at complete removal of the supplied lung area is advisable. In the present case, an aberrant artery perfused the basal segment. We believe that the clinical benefit of preserving segment 6 was limited because pulmonary function was normal and the size of segment 6 was small. Therefore, left lower lobectomy was performed.

**Table 2:** Summarized operative findings in previous cases, including our case (38 reports, 47 patients)

Operative findings	Number of patients
Preoperative treatment	4 (8.5)
Coiling	2 (4.3)
TEVAR	2 (4.3)
Operative procedure	
Lobectomy	26 (55.3)
Segmentectomy	11 (23.4)
Wedge resection	3 (6.4)
Division only of the aberrant artery	7 (14.9)
Treatment method of aberrant artery	
Stapler	24 (51.1)
Stapler+suture/ligation/clip	7 (14.9)
Only suture/ligation/clip	12 (25.5)
Unknown	4 (8.5)
Complication	
None	42 (89.4)
Aneurysm	2 (4.3)
Pneumonia	2 (4.3)
Air leak	1 (2.1)

Values are presented as n (%) unless otherwise indicated.

TEVAR, thoracic endovascular aortic repair

Second, management of aberrant arteries should also be accounted for. **Figure 5B** showed that the need for preoperative inflow control by TEVAR, endovascular coil, or plug embolization should be determined by arterial morphology, particularly aneurysmal changes and large caliber. Utsumi et al.^28)^ reported a case in which aneurysmal aberrant artery division with a linear stapler alone resulted in substantial intraoperative bleeding and subsequently required coil embolization of a residual feeder postoperatively. In contrast, several reports, including ours, have reported safe surgery when TEVAR or coil embolization is performed preoperatively.^34,39)^ Therefore, it appeared appropriate that preoperative management of the aberrant artery should be considered in cases in which the aberrant artery is large in caliber or shows aneurysmal changes. Clear criteria for selecting between TEVAR and endovascular coil or plug embolization have not yet been established. However, Hwang et al.^24)^ reported that their patient ultimately required pulmonary resection because coil embolization alone showed incomplete inflow control. Therefore, as in our case, when the aberrant artery is large in caliber and is presumed to carry a high flow, TEVAR could be a more reliable option for inflow control. As shown in **Fig. 5B**, when preoperative treatment is indicated, anatomical and flow assessments are performed to evaluate the risk of endoleak or re-perfusion. In the presence of complex distal branching, collateral pathways, or presumed high-flow systems, TEVAR is favored to achieve secure proximal inflow control. Otherwise, coil or plug embolization may be selected. The indication for TEVAR is limited to cases with a low risk of Adamkiewicz artery occlusion; embolization is preferred when this risk is considered high to minimize the risk of spinal cord ischemia. Notably, all reported cases of ASABS treated with TEVAR have originated from Japan. This likely reflects differences in healthcare systems, regulatory environments, and institutional practices, rather than a true geographic disparity. Japan is also among the countries with the highest annual TEVAR volumes worldwide, which may contribute to the predominance of Japanese reports. Although TEVAR is not an established or reimbursed indication for this condition, it may represent a feasible option in carefully selected high-risk anatomical settings. According to this framework, TEVAR would be the preferred strategy for inflow control in our patient.

Additionally, aberrant arteries are structurally more fragile than normal arteries, and stump aneurysms can develop after division.^15)^ Accordingly, stump reinforcement such as the application of clips or additional sutures should be considered.

Finally, the optimal interval between TEVAR and pulmonary resection in patients with ASABS should be considered. In the present case, we performed semi-urgent TEVAR to achieve inflow control and then proceeded with pulmonary resection in a staged manner after clinical stabilization because imaging suggested a high risk of rupture of the aberrant artery. Reports of TEVAR-first strategy followed by lung resection are rare; we identified only one comparable case.^34)^ In both cases, second-stage resection was performed after confirming the interruption of inflow and the absence of endoleak. However, a two-stage approach may also permit progressive intraluminal thrombosis, which complicates arterial division. A single-stage approach consolidates anesthesia into one session, yet demands addressing postoperative acute complications and intraoperative heparinization following TEVAR. Whether a single- or two-stage procedure is superior remains uncertain. Therefore, decisions regarding staging should be individualized.

This study has several limitations. First, it described a single case with a relatively short follow-up of 6 months. Therefore, our findings cannot be used as a standard strategy for all patients with ASABS. Second, the decision to perform TEVAR was based mainly on imaging findings rather than on symptoms or hemodynamic instability. In addition, the risk-benefit balance might differ in other clinical settings. Further accumulation of cases and long-term follow-ups are needed to clarify the optimal indications for the TEVAR-first strategy and to evaluate long-term outcomes.

## CONCLUSIONS

We treated a case of ASABS complicated by an aneurysmal aberrant artery with imaging findings suggestive of a high risk of rupture. The TEVAR-first strategy enabled safe left lower lobectomy with division of the aberrant artery. Staged resection that includes preoperative TEVAR can reduce the risk of intraoperative bleeding and permit secure aberrant arterial management. Future studies should define the selection criteria and accumulate data on long-term outcomes.

## SUPPLEMENTARY MATERIALS

Supplementary Figure S1 Chest radiograph at presentation.
The figure showed an abnormal shadow in the left lower lung field.

Supplementary Figure S2 Intraoperative findings.
(a) The figure demonstrated that anaberrant artery was located caudal to the inferior pulmonary vein.
(b) The figure showed the aberrant artery after dissecting the firm adhesion to the inferior pulmonary vein.
(c) The figure demonstrated that we divided the aberrant artery at its origin using a linear stapler (DST Series TA).
(d) The figure showed that polymer ligating clips (Click’a V®, size L, Grena Ltd, UK) were applied on the proximal side of the stapled transection line.

## DECLARATIONS

### Availability of data and materials

All the data and materials relevant to this study are available from the corresponding author upon reasonable request.
