# Supplementary material for: Anomalous Systemic Arterial Supply to the Basal Segments with an Aneurysmal Aberrant Artery Showing Advanced Wall Fragility: A Case Report and Literature Review
Source: Surg Case Rep. 2026 Mar 20;12(1):25-0823. doi: 10.70352/scrj.cr.25-0823 (PMC13033404; doi:10.70352/scrj.cr.25-0823)
Supplement: Supplementary Figure S1 — Chest radiograph at presentation. The figure showed an abnormal shadow in the left lower lung field. [file scr-12-01-25-0823-s001.pdf]

Supplementary Figure 1

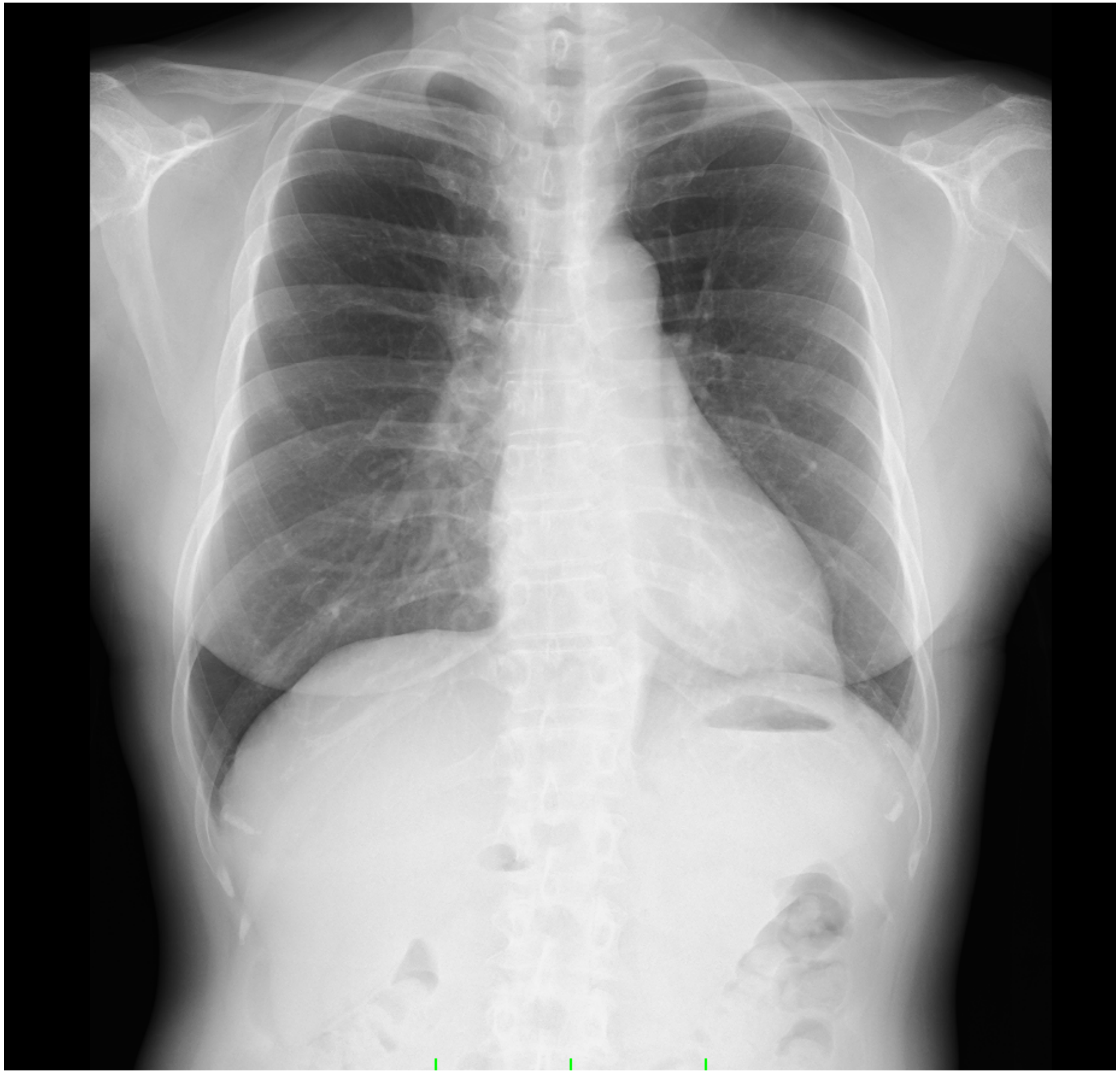

Supplementary Figure S1.

Chest radiograph at presentation.

The figure showed an abnormal shadow in the left lower lung field.

Supplementary Figure S2

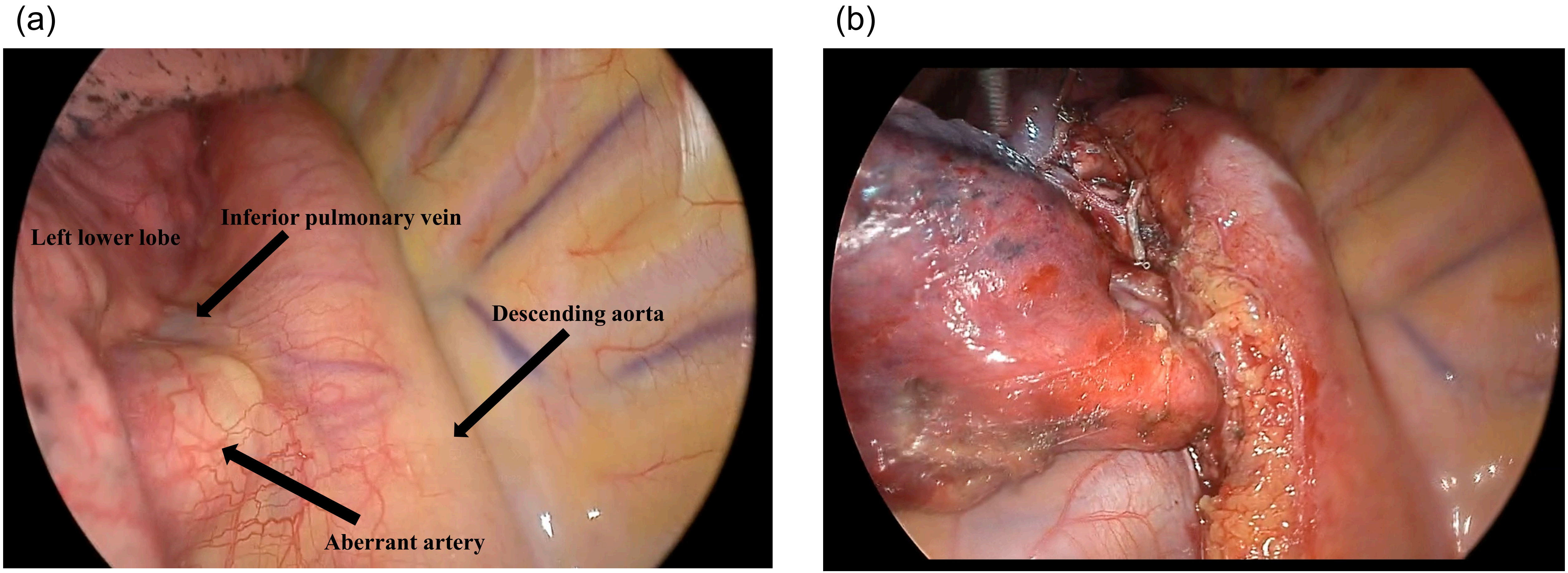

Supplementary Figure S2.

Intraoperative findings.

(a) The figure demonstrated that an aberrant artery was located caudal to the inferior pulmonary vein.

(b) The figure showed the aberrant artery after dissecting the firm adhesion to the inferior pulmonary vein.

Supplementary Figure S2

(c) (d)

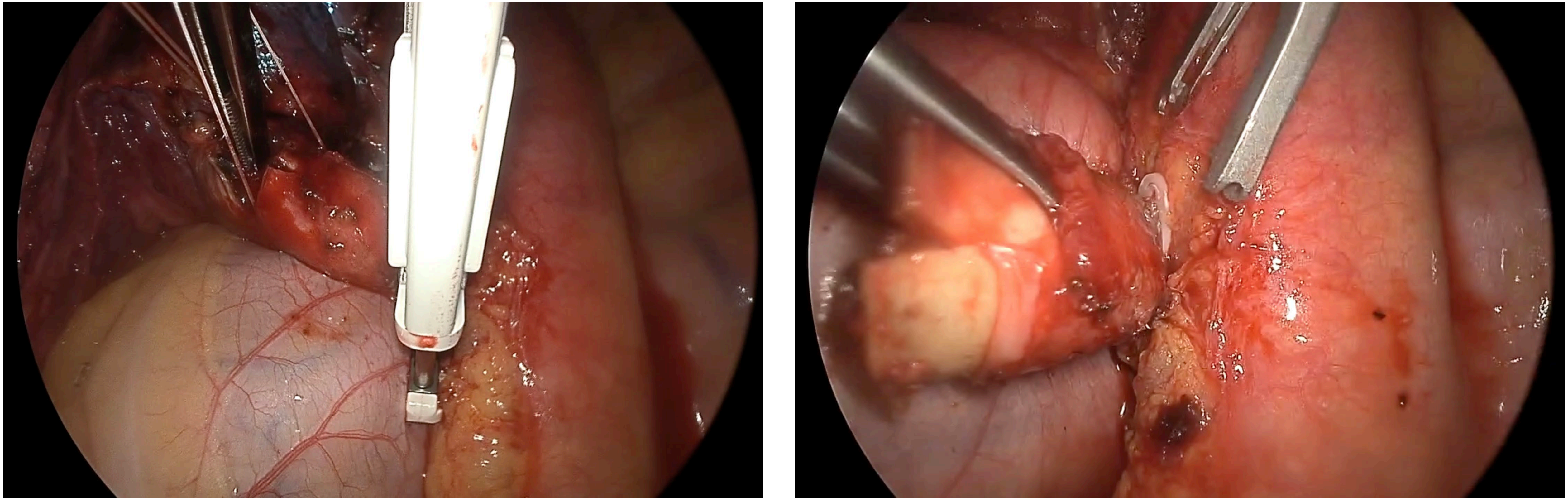

Supplementary Figure S2.

Intraoperative findings.

(c) The figure demonstrated that we divided the aberrant artery at its origin using a linear stapler (DST Series TA).

(d) The figure showed that polymer ligating clips (Click’a V®, size L, Grena Ltd, UK) were applied on the proximal side of the stapled transection line .
